# Pressurized Martian-Like Pure CO_2_ Atmosphere Supports Strong Growth of Cyanobacteria, and Causes Significant Changes in their Metabolism

**DOI:** 10.1007/s11084-015-9458-x

**Published:** 2015-08-21

**Authors:** Gayathri Murukesan, Hannu Leino, Pirkko Mäenpää, Kurt Ståhle, Wuttinun Raksajit, Harry J. Lehto, Yagut Allahverdiyeva-Rinne, Kirsi Lehto

**Affiliations:** Laboratory of Molecular Plant Biology, Department of Biochemistry, University of Turku, Turku, Finland; Centre of Integrated Bioscience for Animal Health and Alternative Energy, Faculty of Veterinary Technology, Kasetsart University, Bangkok, Thailand; Tuorla Observatory, Department of Physics and Astronomy, University of Turku, Piikkiö, Finland

**Keywords:** Biological life support systems, Effects of carbon/nitrogen ratio, Biohydrogen production

## Abstract

Surviving of crews during future missions to Mars will depend on reliable and adequate supplies of essential life support materials, i.e. oxygen, food, clean water, and fuel. The most economical and sustainable (and in long term, the only viable) way to provide these supplies on Martian bases is via bio-regenerative systems, by using local resources to drive oxygenic photosynthesis. Selected cyanobacteria, grown in adequately protective containment could serve as pioneer species to produce life sustaining substrates for higher organisms. The very high (95.3 %) CO_2_ content in Martian atmosphere would provide an abundant carbon source for photo-assimilation, but nitrogen would be a strongly limiting substrate for bio-assimilation in this environment, and would need to be supplemented by nitrogen fertilizing. The very high supply of carbon, with rate-limiting supply of nitrogen strongly affects the growth and the metabolic pathways of the photosynthetic organisms. Here we show that modified, Martian-like atmospheric composition (nearly 100 % CO_2_) under various low pressure conditions (starting from 50 mbar to maintain liquid water, up to 200 mbars) supports strong cellular growth. Under high CO_2_ / low N_2_ ratio the filamentous cyanobacteria produce significant amount of H_2_ during light due to differentiation of high amount of heterocysts.

## Introduction

So far, the life support supplies (oxygenic air, food, water, fuel) have been made available to space mission crews (e.g. the low orbit space stations) by transporting them from the Earth, and by efficient recycling and purification of the gases and the water on board. Hauling of food for possible future missions to Mars is also planned (Cooper et al. 2011), but this would be extremely energy consuming and expensive, at least for extended human stay on such remote station. The most economical and sustainable, and in long term, the only viable means to provide these supplies for the mission crews on Mars is via bio-regenerative systems, by using local resources (In Situ Resource Utilization, ISRU) to drive oxygenic photosynthesis (Cockell 2014; Montague et al. 2012; Perchonok et al. 2012; Verseux et al. 2015).

Most of the resources needed for photosynthesis are available on Mars: the atmosphere contains 95.3 % of CO_2_, water is available either as ice in the underground and in polar areas, or as bound to the minerals, and sun light is available to provide energy for the process. Most of the nutrients (P, K, Mg, Mn, Fe and micronutrients) needed for plant or bacterial growth exist in the Martian regolith (Arvidson et al. 2014; Cockell 2014; Graham 2004; Grotzinger et al. 2014), but some efficient means are needed for their extraction and dissolving into usable form (Olsson-Francis and Cockell 2010). The most striking shortage of the necessary nutrients in Mars is the very low level (2.7 %) (Graham 2004) of nitrogen in the atmosphere, and its near absence from the regolith, meaning that an extra supply of this nutrient needs to be brought over from the Earth.

The local conditions on Martian surface are hostile to terrestrial organisms. The most detrimental factors there are the strong UV radiation (200–300 nm, UV flux), low air pressure (varying between 5 and 10 mbar), and low, but widely varying temperatures (Cockell 2014; de Vera et al. 2010; Horneck et al. 2010). Mean surface temperature is −60 °C, the maximal daily temperature variation range is 40–70 °C, but during the warmer season the maximum daily temperatures raise at the equator up to +20 °C (Graham 2004). If protected against damaging radiation, the resting forms of some microbial species survive in such harsh conditions (see e.g. special edition: The Expose E-mission. Astrobiology, May 2012, 12(5), 373–528). The most durable photosynthetic eukaryotes, i.e. *Xanthoria elegans* lichens can even show photosynthetic activity in the low temperature regimes which allow water saturation in low pressures (down to 10 mbar), when supplied with CO_2_ and suitable level of light (photosynthetically active radiation, PAR, in the range of 100 μE) (de Vera et al. 2010). Still, these conditions prevent the long term survival and active production of any photosynthetic organisms in the open Martian environment.

Therefore, the photosynthetic organisms need to be adequately shielded against UV and hard cosmic radiation, and contained in fully enclosed facilities to protect them against low pressure and desiccation. A tight enclosure is also required for maintaining the produced oxygen. All assimilated nutrients need to be efficiently recycled in the closed systems to make best use of these supplies, and to protect the environment from contamination. Particular care needs to be taken of the maintenance of ammonium waste, which in the absence of oxygen cannot be oxidized to nitrates and is easily lost via evaporation. Extensive research to this end has been done for example in the Microbial ecology of the closed artificial ecosystem (MELiSSA) program, coordinated by the European Space Agency (ESA), aiming to resolve how all organic wastes (gas, liquid and solids) can be completely recycled in a closed loop of multiple compartments and bioreactors (Hendrickx et al. 2006; Hendrickx and Mergeay 2007).

In comparison to these fully controlled culture units and bioreactors, a more economical bio-regenerable life support system could be obtained in conditions that are as close to the outside parameters as possible. Particularly, maintenance of the cultures at minimal suitable pressures would allow lower construction weight and cost, and also minimize leakage of gases and the risk of the escape of organic matter (Lehto et al. 2006; Richards et al. 2006). Still, the conditions need to be adjusted so that they allow efficient or at least adequate growth of the cultivated organism, depending on their (minimal) demands and biological adaptation potential.

Cyanobacteria are simple but highly efficient photosynthetic organisms, and some of them thrive in the most varied and extreme habitats on Earth, e.g. in the dry deserts of Antarctic, or within the ices of high Arctic seas (Scalzi et al. 2012); (Wierzchos et al. 2006). Many cyanobacteria survive also in space conditions when protected from UV-radiation (Billi et al. 2013; Olsson-Francis et al. 2010). At least some cyanobacterial species are durable against high CO_2_ concentrations, and upon gradual adaptation can survive even in 100 % CO_2_ atmosphere, in 101 kPa (1 atm) pressure and can use elevated CO_2_ concentrations as carbon fertilizer, with increased growth in moderately high partial pressures of CO_2_ (Olson 2006); Thomas et al. 2005; Kanervo et al. 2005). As cyanobacteria are adaptable to the harsh and restrictive growth conditions, they could form the first photosynthetic component of the bio-regenerable life support system on Mars. Some cyanobacterial species also grow as lithotrophs, extracting their nutrients directly from the rocky minerals, and can thus be used as bio-leaching agents to release nutrients from Martian basalt (Montague et al. 2012; Olsson-Francis and Cockell 2010). Many cyanobacteria are efficient biomass and oxygen producers, and some species produce edible biomass. Some cyanobacteria also can produce hydrogen (H_2_) under light, as a by-product of the nitrogen fixation by nitrogenase enzyme activity (Masukawa et al. 2012). This process is induced in low nitrogen conditions (reviewed in (Allakhverdiev et al. 2009; Dutta et al. 2005; Quintana et al. 2011; and Raksajit et al. 2012). Thus, they also provide a new possibility for renewable bioenergy production, particularly in atmospheres of Martian-like composition with very low N_2_ content. A more thorough discussion of the possible uses of different cyanobacteria as biological life support organisms on Mars can be found at Verseux et al. (2015).

Here we have tested the growth of selected cyanobacterial species in variable CO_2_ concentrations and partial pressures. First, we tested the effect of different partial pressures of CO_2_, either as mixed in normal air or as pure (100 %) CO_2_, on growth of *Synechocystis* sp. PCC 6803, a non-nitrogen fixing unicellular model cyanobacterial species. Further on, we tested the effect of the pure CO_2_ atmosphere on the growth of filamentous non-heterocystous *Arthrospira platensis*, and on the growth and H_2_ photoproduction of the N-fixing filamentous heterocystous *Anabaena cylindrica. A. platensis* and *A. cylindrica* were used here as the test species due to their high relevance for bio-regenerable life support systems: *A. platensis* produces a highly nutritious, edible biomass (Lehto et al. 2006) and is also a fairly efficient H_2_ producer (Raksajit et al. 2012). *A. cylindrica* is known as an efficient H_2_ producer (Dutta et al. 2005) and also as an efficient nitrogen fixer and a lithotroph, able to grow on mere powdered basalt in water (Olsson-Francis and Cockell 2010). While this work aims at testing how the Martian-like atmospheric composition affects the growth and metabolism of these different types of cyanobacteria, it also provides new approaches for enhancing cyanobacterial biohydrogen production in terrestrial applications.

## Methods

### Test Species and Culture Conditions

The cyanobacterial species used in this study, i.e. *Synechocystis* sp. PCC 6803, *Arthrospira platensis* (PCC8005), and *Anabaena cylindrica* (PCC 6309); all originate from the Pasteur culture collection (Paris, France). These strains have been preserved either as frozen in −70 °C, or as continuously cultured laboratory stocks in their optimal temperature and light regimes at our home laboratory, at the department of Biochemistry, at University of Turku. *Synechocystis* sp. PCC 6803 and *A. cylindrica* were grown in BG-11 medium and *A. platensis* in Zarrouk medium. The constant light and temperature conditions for these species were 50 μmol photons m^−2^ s^−1^ PAR and 32 °C, 50 μmol photons m^−2^ s^−1^ and 25 °C, and 70 μmol photons m^−2^ s^−1^ and 32 °C, respectively. For maintenance, all these cultures were grown under agitation (typically at 100 rpm).

**Fig. 1:**
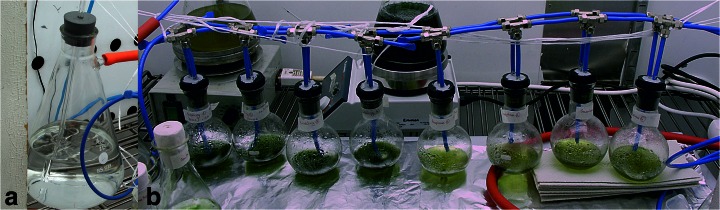
The vacuum line set-up where the flowing gas is first saturated with water vapor (*left*) and then lead into the culture bottles. The air flow is regulated with a rheostat and the pressure inside the whole closed system by a pressure gauge, and a vacuum pump (vacuubrand)

For testing the cell growth in different CO_2_ concentrations and pressure conditions, the cultures of each species were set up in the same temperature and light conditions as stated above, in round-bottomed flasks, connected to a fully closed aeration line (Fig. 1). Air to this line was drawn from a 99.99 % CO_2_ bottle (AGA, from here on, referred as 100 % CO_2_), or from a container where the CO_2_ was mixed into ambient air in desired concentrations, adjusted with a CO_2_ regulator (Vaisala GMM221 probe). The gas flow was regulated by a gas flow regulator, and connected to a vacuum pump and a pressure gauge to maintain the pressure at desired level. The *Synechocystis* cells were grown under different ratios of CO_2_ mixed to ambient air (20, 10, 5, 0.4 % CO_2_) under ambient (about 1000 mbar) pressure, or in 100 % CO_2_ gas under pressures varying from 50 to 400 mbars; *A. platensis* cells were grown likewise in 100 % CO_2_ gas under different pressures, and *A. cylindrica* cells were grown in 100 % CO_2_ under 100 mbar pressure, and also in 10 % CO_2_ mixed into ambient air, under ambient pressure. To reduce the evaporation of the cultures under low pressure conditions, the incoming gas was saturated with water by leading it to flow through a water flask before it entered into the culture vials (Fig. 1).

*Synechocystis* and *A. platensis* growth tests were conducted in 6 liter flasks, in a culture volume of 350 ml, stirred at 100 rpm rate, as described earlier (Kanervo et al. 2005). The cultures were typically run for a 72 h culture period, to maintain the cultures in linear growth phase. Atmospheric treatments for the *A. cylindrica* cultures were done in similar low-pressure gas line, but in smaller (250 ml) flasks, within 50 ml culture volume (Fig. 1). In this set up, the cultures were either mixed by bubbling, or were not mixed. These cultures were run for 7 days before harvesting. The cell densities were quantified by measuring the optical density at 750 nm (OD_750_) before and after the growth period.

Cultures were typically inoculated into a cell density of OD_750_ 0.2, and the cell growth was determined as the relative increase of the OD_750_ in ratio to the growth of control cultures at the ambient conditions, i.e. the growth of each culture was first determined by dividing its final OD_750_ by its initial OD_750_, and then its relative growth was determined by dividing its calculated growth by the average calculated growth of the control cultures. The cultures were conducted using either three or four biological replicates, and each of the experiments was repeated at least two times, with similar results from the experiments. Control cultures were grown in ambient air, in the same light and temperature conditions as the test cultures. Experimental cultures were either mixed by stirring at 100 rpm, or by bubbling of the gas into the culture medium, or grown stationary.

### Measurement of the Hydrogen and Oxygen Yields in Head Space of the Culture Vials

The hydrogen and oxygen production potential of *A. cylindrica* cells was determined as described previously (Leino et al. 2014). Cell samples cultivated either in ambient air, or in the 100 % CO_2_ atmosphere in 100 mbar pressure for 7 days were harvested by pelleting, washed twice with BG-11_0_ (BG-medium without combined nitrogen), and adjusted to 5 mg chlorophyll *a* per ml concentration in BG-11_0_ medium. Five milliliter samples of these cell suspensions were taken into air tight 20 ml glass vials. The vials were thoroughly flushed with argon to create anaerobic condition in the head space of the vials. The vials were tightly sealed with rubber septa, supplemented with 10 % CO_2_ when needed, and incubated overnight with constant shaking and illumination (150 μmol photons m^−2^ s^−1^) at 25 °C. The following day, 150 μl of air from the head space in each vial was drawn with a gas-tight syringe (Hamilton Co.) and plunged into a gas chromatograph (Perkin Elmer Clarus 500, a Molecular Sieve 5 A, 60/80 Mesh column) to detect and quantify the gases produced, as described earlier (Leino et al. 2012, 2014; Raksajit et al. 2012).

### Microscopy

The samples for microscopy were prepared as wet mounts (unstained) to observe the specimens in their natural condition. Bright-field images were captured using light microscope (Orthoplan, Leitz) equipped with a digital camera (Leica DFC 420C) using 10 × objective. Heterocysts were distinguished by their thick cell envelope, and vegetative cells between two heterocysts along the filaments were counted to determine the heterocyst frequency. Multiple filaments from three biological replicate samples were scored for each experimental condition.

## Results

### Effects of High CO_2_ Concentration and of Culture Mixing on the Growth of Different Cyanobacteria

Testing the effects of the low pressure atmospheres with Martian-like composition (anoxic, high CO_2_ content, low N) as an environment for photosynthetic organisms, in terrestrial conditions, must be conducted in vacuum-tight containers. In such closed system the CO_2_ will be quickly depleted, unless it is continually replenished by a controlled gas flow. This flushing of the culture vials with CO_2_ also allows maintenance of these photosynthesizing systems under minimal oxygen conditions, simulating the Martian atmospheric composition (except for the N_2_ which we here provide solely from the culture medium). In these conditions the culture liquid may be quickly lost through evaporation, but this can be prevented, to some extent, by saturating the gas flow with water. Suitable total gas pressures and flow can then be applied to reduce the water evaporation to acceptable level, and to produce desired supply of the gas. This system also allows testing the effects of anoxic, high CO_2_ containing and nitrogen depleted atmospheres on the growth, and on the H_2_ photoproduction of different cyanobacterial species.

**Fig. 2:**
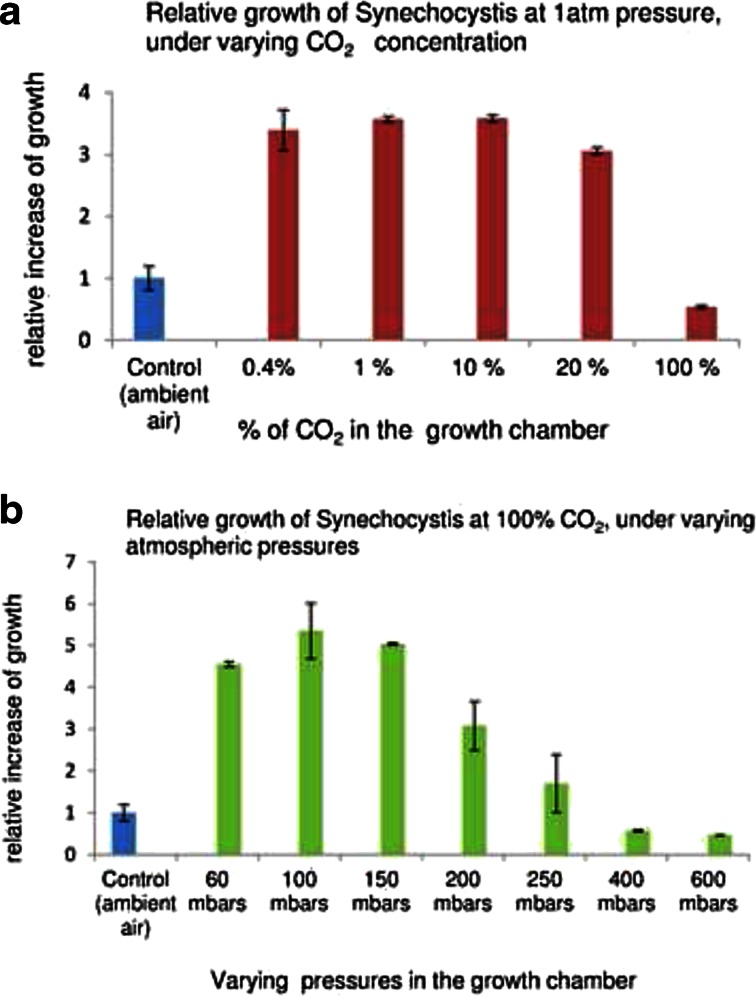
Relative growth rate *Synechocystis* sp. PCC 6803 cells grown under 1 atm (ambient) air pressure, in varying CO_2_ concentrations (**a**) or in 100 % CO_2_ under variable pressures, (**b**), for 3 days, calculated as the relative increase of OD750, in ratio to the relative increase of the control cultures grown in ambient air. All cultures were stirred at 100 rpm rate

Monitoring of the growth of the model cyanobacterial species, *Synechocystis* sp. PCC 6803 in high CO_2_ concentrations and under different air pressures, under 3-day culture periods, revealed that the growth of this strain is affected mostly on the partial pressure of CO_2_ supply. All CO_2_ concentrations elevated above of the ambient concentration (0.04 %), up to 20 %, as mixed in air, or as pure CO_2_ supplied in pressures ranging from 50 mbar (the lowest pressure which still allowed stable maintenance of liquid culture medium in our experimental system) up to 200 mbar (corresponding to 20 % of CO_2_ mixed in ambient air) enhanced strongly the growth. CO_2_ elevated up to 0.4 % in air saturated the carbon assimilation machinery, and the same maximal growth response (about 3.5-fold increase, as compared to ambient air) was obtained between 0.4 and 20 % of CO_2_ mixed in air. Even stronger growth increase (about 5-fold) was obtained when the cells were supplied with pure CO_2_, under pressures up to 150 mbar (Fig. 2). This increase could be caused by the absence of the photorespiration (i.e. by the oxygenation of the enzyme RuBisCO) in the anoxic conditions (Raven et al. 2012). The photorespiration should not play significant role in cyanobacteria, where the CO_2_ concentration around RuBisCo enzyme is controlled by the cellular Carbon concentration mechanism (Kaplan et al. 1991), but it may still cause some reduction of carbon fixation in oxic atmospheres. It is also possible that the low pressure per se enhanced the gas exchange and carbon availability in the culture fluid, or in cellular cytosol, as described by Richards and co-workers (Richards et al. 2006). CO_2_ levels above 400 mbar increasingly inhibited the cell growth, which effect might be reduced by gradual adaptation of the cells to these high CO_2_ concentrations (Thomas et al. 2005).

**Fig. 3:**
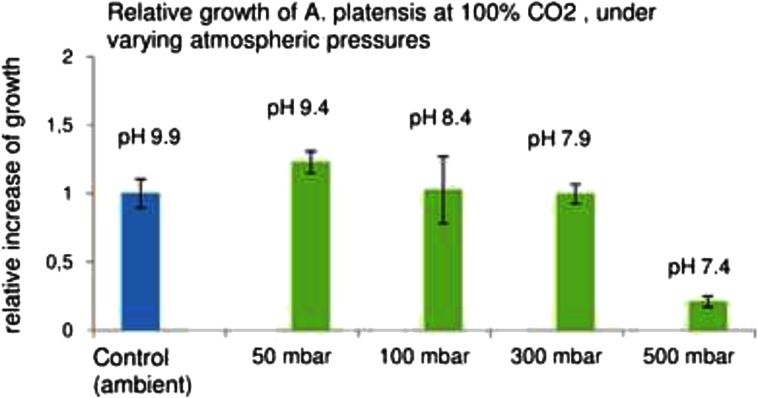
Relative growth rate *A. platensis* cells grown under varying air pressure, in 100 % CO_2_ for 3 days, calculated as the relative increase of OD 750 in ratio to the relative increase of the control cultures grown under ambient air. All cultures were stirred at 100 rpm rate. Final pH of the cultures is marked above the bars. The initial pH of the cell suspensions, prior to the culturing, was 9.38

Next, the effect of 100 % CO_2,_ supplied under different pressures, was tested on the growth of *A. platensis*. Here the CO_2_ supplied at 50 mbar pressure enhanced the growth, although only by 25 %, as compared to the growth of control cultures in ambient air (Fig. 3). This low response to the CO_2_ fertilizing may be due to the presence of high level of sodium bicarbonate (0.2 M) in the Zarrouk medium, and due to the significant decrease of the culture medium pH in these treatments.

### Growth of *Anabaena cylindrica*, and Induction of its H_2_ Photoproduction Under High CO_2_, Low Nitrogen Conditions

**Fig. 4:**
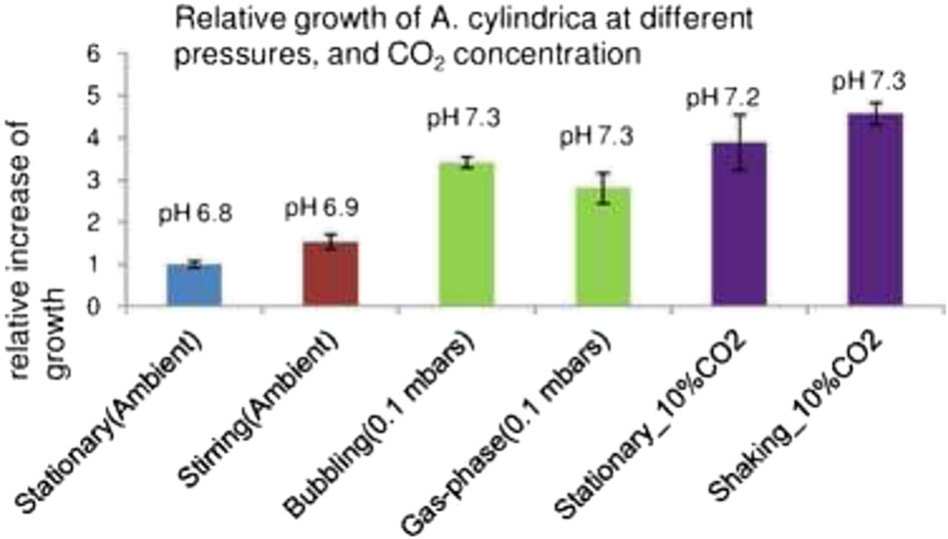
The growth rate *A. cylindrica* cells grown either stationary or with stirring under ambient air (*blue* and *red bars*), or in ambient air with 10 % CO_2_ enrichment (*violet bars*), or, stationary or with bubbling under 100 mbar pressure in 100 % CO_2_ (*green bars*), for 7 days

As the 100 % CO_2_ supplied in 100 mbar pressure was found to support strong cyanobacterial growth, we test the effect of these conditions on the growth and the H_2_ photoproduction potential of the nitrogen fixing species *A. cylindrica.* This strain was cultivated under 100 % CO_2_, 100 mbar pressure, in the rich BG11-medium for 7 days, to allow the cultures to enter stage where the nitrogen supply became limiting for the cell growth. These conditions produced a total of 3-fold increase of the cell growth, as compared to the growth in ambient air (Fig. 4). However, even higher growth (4-fold) was obtained in 10 % CO_2_ concentration in air, under the ambient pressure, where the cells also had access to the unlimited atmospheric nitrogen. Furthermore, the mixing of the liquid cultures, either by agitation, or by bubbling of the CO_2_ flow into the medium (Fig. 1), was found to slightly enhanced the growth. This indicated that agitation may improve the gas exchange in the culture medium, or enhance growth by avoiding shading of the culture, but this treatment effect was not statistically significant (Fig. 4).

**Fig. 5:**
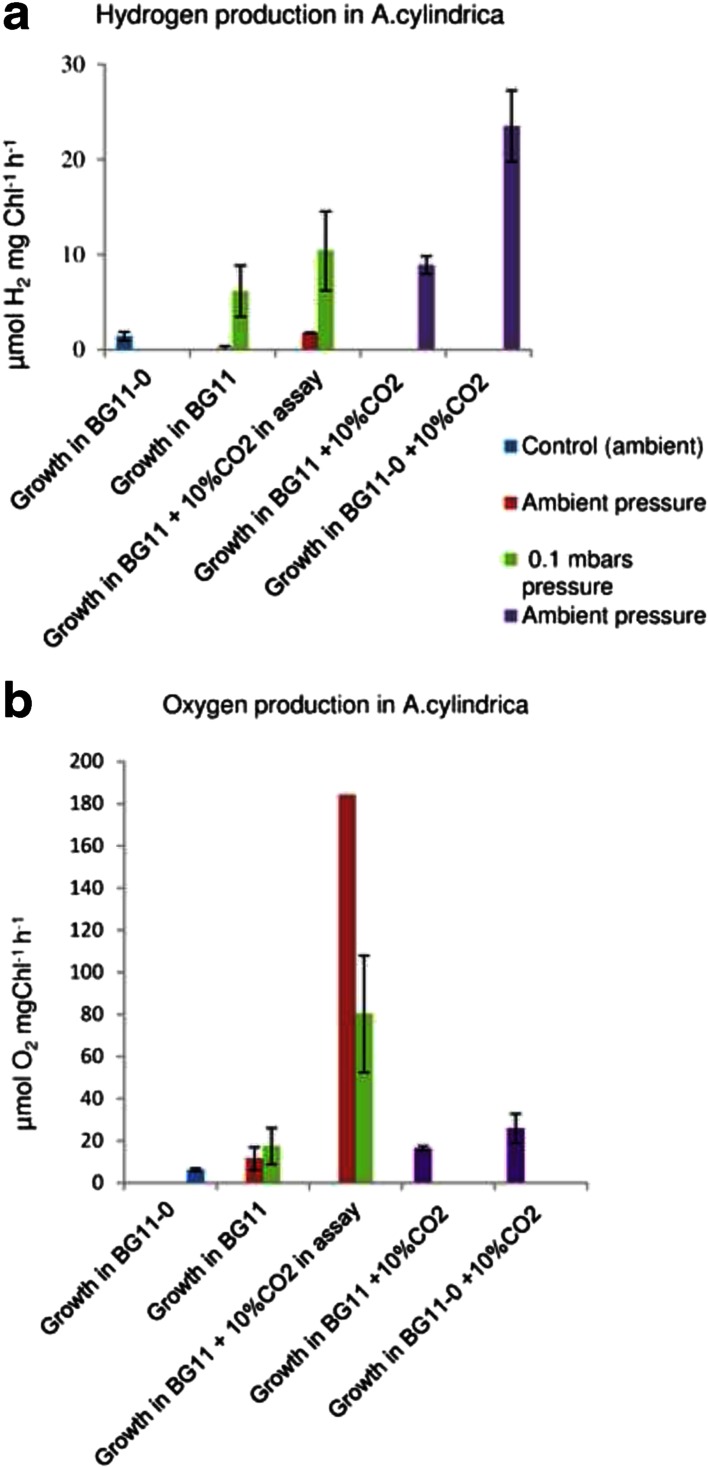
Hydrogen (H_2_) production (**a**) and oxygen (O_2_) production (**b**) of *A. cylindrica* cells, measured as μmols of H_2_ or O_2_ produced / mg Chl *a*, in 1 h, after 7 days cultivation of cells, using either ambient air pressure, or 100 mbar pressure, and using different CO_2_ concentrations both in the culture vials, and in the incubation vials (Ar + 10 % CO_2_) during the gas production, as indicated in the figure. The green bars indicate cultures grown in 100 mbar pressure in 100 % CO_2_, simulating cultures in pressurized Martian atmosphere. The red and the blue bars indicate control cultures grown in ambient air, and the purple bars indicate cultures grown in ambient air supplemented with 10 % of CO_2_

*A. cylindrica* grown in BG11 medium, at ambient air and pressure (control cultures) do not show any, or only very low H_2_ production (0.2 μmol H_2_ mgChl *a*^−1^ h ^−1^) (Fig. 5). This is not a surprise, since H_2_ production in this strain is mostly originated from nitrogenase, which is a heterocyst specific enzyme and is not present in the filaments grown in the presence of combined N (BG11) (Masukawa et al. 2010). In the absence of combined nitrogen in the growth medium (BG11_0_) some vegetative cells differentiate to heterocysts which, due to thick cell wall and high respiratory activity provide a unique micro-oxic condition for the O_2_ sensitive enzymes related to N_2_ fixation (Meeks and Elhai 2002). In line with this, *A. cylindrica* grown in the BG11_0_ medium, lacking combined nitrogen source, under ambient air and pressure conditions, produced H_2_ at a rate of about 1.7 μmol H_2_ mgChl*a*^−1^ h ^−1^. Interestingly *A. cylindrica* cultures grown in BG11-medium, in low pressure (100 mbar), in 100 % CO_2_ conditions, produced H_2_ at level of 6.1 μmol H_2_ mg Chl *a*^−1^ h ^−1^, i.e. enhanced by about 30-fold, as compared to the cells grown in rich BG11-medium, in ambient air, and also about by 4-fold, when compared to cells grown in BG11_0_ medium, in ambient air (Fig. 5a).

To clarify whether such an enhancement in H_2_ production was due to excess CO_2_ amount or due to applied low pressure, we next cultivated cells for 7 days in both BG11 and BG11_0_ medium under ambient air enriched with 10 % CO_2_ (Fig. 5a). Under these conditions the cells grown in BG11 (with adequate combined nitrogen added) produced hydrogen approximately at the same rate as cells in 100 mbar pressure in 100 % CO_2_ (no significant difference), but the cells grown in BG11_0_ showed the maximal observed H_2_ production (25.9 μmol H_2_ mgChl*a*^−1^ h^−1^), or a 15-fold higher level than what was observed in the cells grown under ambient air and pressure, in BG11_0_.

These results strongly suggested a positive effect of high CO_2_ supply on H_2_ photoproduction rate in *A. cylindrica.* Since application of high amount of CO_2_, under low pressure, stimulated H_2_ photoproduction even in the cells grown under BG11 medium, next we monitored the possible effect of 100 % CO_2_ and low pressure on heterocyst formation. In correlation with the high H_2_ photoproduction, we found that the number of heterocysts was strongly increased in the cultures that were grown in high CO_2_ atmospheres, as compared to cultures grown in ambient air. Filaments grown in 100 % CO_2_, in 100 mbar pressure, in non-mixed cultures contained an average of 12 (+/− 3) cells, and in the bubbled cultures an average of 13 (+/− 4) cells (difference not significant) between adjacent heterocysts, while the filaments grown in the ambient air contained an average of 27 (+/−14) cells (Fig. 5). The occurrence of heterocysts in the control cultures indicated that also these cultures had already depleted the combined Nitrogen from their BG11 culture medium during the 7 day culture period. The higher number of heterocysts in the filaments in the low pressure, 100 % CO_2_ conditions indicated higher nitrogen deficiency and higher nitrogen fixing activity in these *Anabaena* cultures, as can be expected to happen in response to stronger biomass accumulation, leading to stronger depletion of the combined nitrogen from the growth medium (Adams and Carr 1981; Meeks and Elhai 2002; Wolk et al. 1994).

**Fig. 6:**
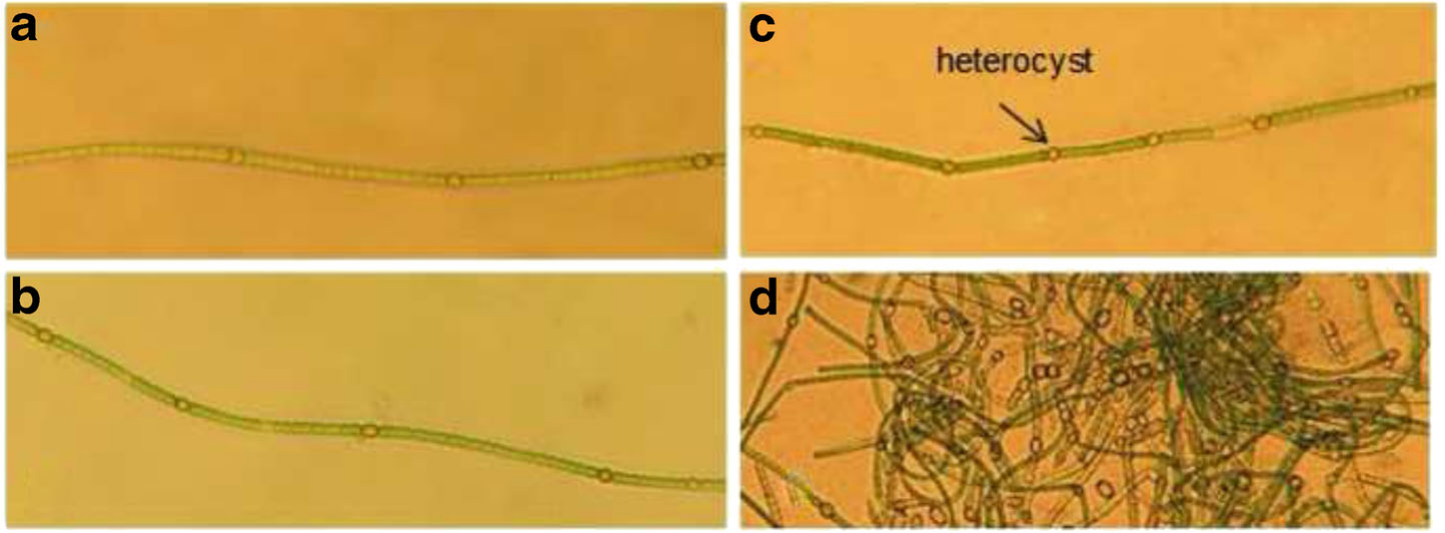
Occurrence of heterocyst in cultures after a growth of 7 days in the following conditions: Control cultures (stationary, ambient air) (**a**); cultured under 100 % CO_2_, 100 mbar, with bubbling, (**b**); cultured under 100 % CO_2_, 100 mbar, stationary (**c**); cultured under 10 % CO_2_ –enriched ambient air (**d**)

Supply of 10 % CO_2_ under ambient air and pressure induced a massive increase of the heterocysts in cells grown in BG11_0_, including occurrence of heterocysts in doublets and strings (Fig. 6d). This indicated that the nitrogen fixing machinery was maximally induced under these conditions where the increased CO_2_ supply maintained strong biomass production, which was solely supported by the biological N_2_ fixation. After transfer to N-depleted incubation conditions this whole machinery was directed into strong H_2_ photoproduction.

All cell samples treated under these different atmospheric conditions produced oxygen at approximately equal level (Fig. 5b), indicating that they all remained in functional photosynthetic condition. Interestingly, the only factor which significantly increased the oxygen production rate both of the control cells and of the 100 % CO_2_ –treated cells was again the enrichment of head space gas (Argon) with 10 % of CO_2_ during the incubation period for the gas production (Fig. 5a and b). This significant enhancement of both the O_2_ and H_2_ production under 10 % CO_2_ shows that the photosynthetic gas production is directly dependent on the availability of CO_2_, as the photo assimilated carbohydrates provide the chemical energy to the electron transfer reactions.

## Discussion

Producing photosynthetic organisms on Martian surface, solely relying on local Martian resources and recycling of nutrients, would be highly useful for providing bio-regenerable life-supports materials for the crews of future Mars missions. The local conditions are too harsh for any intensive bio-production on the Martian surface, but production is feasible in closed growth compartments, which allow adjustment of temperature, radiation shielding, and adequate pressure to maintain water in liquid form. As the conditions still are harsh and deviating from the terrestrial conditions, selected cyanobacteria would be the most suitable pioneer species to be cultured in such demanding conditions, for initial bioleaching of nutrients, nitrogen fixing and oxygen production.

Although the physical growth conditions could be otherwise adjusted as needed, the local atmosphere, with 95.3 % CO_2_ and 2.7 % nitrogen, under adjusted pressure, would serve as the raw material for carbon and nitrogen bio-assimilation. The ratio and the partial and total pressures of these gases are very different from that on Earth, and would strongly affect the cellular growth and metabolism. These effects are still very poorly known, mostly due to their lack of practical relevance in terrestrial conditions, and due to their difficult technical application under terrestrial atmospheres. Particularly, the effect of air pressure has not been considered as an essential question for terrestrial biology as no biological niches occur below 330 mbar pressure, at the top of the Mt. Everest (Fajardo-Cavazos et al. 2012). Thus, this has been studied only as a space-research related parameter (de Vera et al. 2010; Richards et al. 2006).

The carbon and nitrogen assimilation reactions are closely inter-connected in nitrogen-fixing cyanobacteria, and dependent on the gas composition of the prevailing atmosphere. In terrestrial atmospheres CO_2_ is a limiting substrate for the net carboxylation reaction, and O_2_ competes with CO_2_ as the substrate of the RuBisCO enzyme. Atmospheric N_2_ provides a non-limiting substrate for nitrogen fixation reactions, but these reactions are very energy demanding and are solely dependent on carbohydrates (energy) provided by the photosynthetic cells in the filaments. The conditions are completely opposite in the ambient Martian atmosphere, where CO_2_ is abundant, nitrogen strictly limiting for the bio-assimilation process, and O_2_ does not interfere either with the carbon assimilation or nitrogen fixation reactions. A major difference is also the low atmospheric pressure (c. 7 mbar), which prevents maintenance of liquid cultures, and therefore demands use of pressurized gas conditions.

Results of this work indicate that the CO_2_ and nitrogen supply, and presence or absence of oxygen affect the cyanobacterial growth in complex and interdependent manner. All the tested three species grew well under the low pressure conditions, as long as water evaporation was prevented by moisturizing of the gas flow, CO_2_ supply was maintained on adequate level by continuous replenishing, and adequate nitrogen was provided for the growth period either from the culture medium or from the air (for nitrogen fixing species). Pure CO_2_ atmospheres were tolerated by all these species, and it enhanced the growth in species-specific manner under lower pressures, but become gradually inhibitory if applied on too high levels. In sensitive species (*A. platensis*) the growth inhibitions appears to be related to excessive acidification of the culture medium-which again is determined between the enhanced cell growth (reduces acidity), and the dissolving of CO_2_ (increases acidification). This result is in accordance with published results indicating that cyanobacteria grow to variable extend under high CO_2_ conditions (Thomas et al. 2005), and even higher plants adapt to grow under low atmospheric pressures when supplied with adequate CO_2_ (Richards et al. 2006).

The high CO_2_ and low pressure conditions, simulating pressurized Martian atmosphere was found to affect significantly the H_2_ photoproduction potential of *A. cylindrica* species. The induced imbalance in the C/N supply served to maintain high biomass production for the limited culture period that was used here, and lead to strong induction of the N_2_ fixing machinery, as indicated by high production of the heterocysts. In absence of atmospheric N_2_ (as in the simulated Martian atmosphere, or in experimental anoxic H_2_ production under Argon gas), the nitrogenase enzyme allocates all electrons exclusively to H_2_, and thus leads to enhanced H_2_ production (Masukawa et al. 2010, 2012). This process can be maintained on high productive level even for long periods if the cell biomass growth is arrested e.g. via immobilization to alginate films, as described earlier (Leino et al. 2012), and maybe also by severe depletion of the nitrogen supply, as would occur in Martian-like environment. Adequate supply of CO_2_ (e.g. on 100 mbar level, as in these experiments) in the incubation environment allows the maximal transfer chemically fixed energy into the electron transfer process, and evolution of both O_2_ and H_2_.

Still, in our experimental setting the strongest induction of the heterocyst formation and of the hydrogen photoproduction potential was observed in cultures that were grown in BG11_0_ (nitrogen depleted) medium, under ambient pressure, in normal air supplemented with 10 % CO_2_. This CO_2_ fertilizing effect, with simultaneous unlimited supply of atmospheric nitrogen enhanced both the carbon assimilation and nitrogen fixing machineries to their maximal levels. These machineries lead to maximal H_2_ photoproduction as soon as the cells were transferred into nitrogen-free, anaerobic incubation conditions.

Altogether, we have tested the effects of Martian-like atmospheric composition on the growth and metabolic pathways of selected cyanobacteria. In our experiments these parameters were tested only by end-point measurements, not as real-time response curves, because the cultures could not be opened for sampling during the treatments. Thus the results are only indicative of the effects caused by these conditions, and show only the direction of cellular adaptation and response to these conditions, under culture periods of either 3 or 7 days. Real-time optical growth measurements are needed to measure accurately these parameters. Long-duration cultures are needed to reveal how the cells adapt to these variable parameters on long term time scales. The minimal levels and suitable ratios of the carbon and nitrogen needed for the maintenance of cell growth and productivity need to be measured in future experiments.
